# Enrichment of Total Flavonoids and Licochalcone A from *Glycyrrhiza inflata* Bat. Residue Based on a Combined Membrane–Macroporous Resin Process and a Quality-Control Study

**DOI:** 10.3390/molecules29102282

**Published:** 2024-05-12

**Authors:** Xiaoxia Wang, Zhou Zhang, Yun Wang, Yayi Wu, Li Miao, Yue Ma, Lihua Wei, Wen Chen, Hong Li

**Affiliations:** Key Laboratory of Xinjiang Phytomedicine Resource and Utilisation, Ministry of Education, School of Pharmaceutical Sciences, Shihezi University, Shihezi 832002, China; 20212115046@stu.shzu.edu.cn (X.W.); zhangzhou@stu.shzu.edu.cn (Z.Z.); wangyunn@stu.shzu.edu.cn (Y.W.); 20201007256@stu.shzu.edu.cn (Y.W.); 20212115035@stu.shzu.edu.cn (L.M.); 20212015013@stu.shzu.edu.cn (Y.M.); 20212015014@stu.shzu.edu.cn (L.W.)

**Keywords:** *Glycyrrhiza inflata* Bat. residue, total flavonoids, licochalcone A, UPLC fingerprint, quality control

## Abstract

*Glycyrrhiza inflata* Bat. produces a lot of licorice waste after water extraction, which also retains abundant total flavonoids (TFs) and licochalcone A. However, licorice residue is often wasted due to the lack of good utilization of resources in practical applications. This study first screened the optimal membrane pore size and resin type and then explored the mechanism and conditions of the adsorption of TFs on the resin. Then, different combinations and sequences of membrane and macroporous resin (MR) methods were investigated. It was found that using the membrane method for initial purification, followed by the MR method for further purification, yielded the best purification results. Next, response surface methodology was utilized to investigate the resin’s dynamic desorption conditions for TFs. Finally, the TF purity increased from 32.9% to 78.2% (2.38-fold) after purification by a combined membrane–MR process; the purity of licochalcone A increased from 11.63 mg·g^−1^ to 22.70 mg·g^−1^ (1.95-fold). This study verified the feasibility of enriching TFs and licochalcone A from licorice residue using a membrane–MR coupling method. In addition, a quality-control method was established using a fingerprinting method on the basis of ultrahigh-performance liquid chromatography (UPLC) to ensure the stability of the enrichment process.

## 1. Introduction

*Glycyrrhiza inflata* Bat. is a licorice species recorded in the Chinese Pharmacopoeia (2020 edition). It is distributed mainly in the northwestern regions of Xinjiang and Gansu in China [1]. It has been shown that *G. inflata* Bat. is rich in triterpenoid saponins, flavonoids, coumarins, polysaccharides, and phenolic constituents, which have anti-inflammatory, immunomodulatory, neuroprotective, antimicrobial, and antiviral activities [2]. Companies that produce and process licorice tend to extract and separate glycyrrhizic acid from licorice mainly by boiling and extraction. In this way, many fat-soluble flavonoid components are retained in the medicinal residue of licorice. These total flavonoids (TFs) have antibacterial, hypolipidemic, hypoglycemic, and antioxidant effects [3].

Licochalcone A stands as one of the foremost active constituents of TFs and is unique to *G. inflata* Bat [4]. Studies have shown that licochalcone A has demonstrated the ability to demonstrate anti-inflammatory, antimicrobial, and antioxidative characteristics. Licochalcone A holds significant promise in treating and preventing illnesses [5]. Studies have shown the residue of *G. inflata* Bat. to be the best source of licochalcone A [6]. However, licorice residues are often used to make fertilizers, cardboard, or direct landfills, leading to the squandering of resources and detrimental impacts on the environment [7]. Scholars have used macroporous resins (MRs) to separate and purify the flavonoids present in licorice [8,9]. Acid–base precipitation has been used primarily for the industrial production of licorice extracts. However, the complexity and expense of this process (as well as the low purity of TFs in licorice and licochalcone A in the extract [10]) have hampered production. Therefore, developing and utilizing licorice residue efficiently in an environmentally friendly (“green”) manner is a major challenge.

The traditional methods of separating and purifying active substances from traditional Chinese medicine (TCM) formulations are acid–base precipitation, salt precipitation, and clarifying agents. However, their disadvantages are high consumption of energy, low efficiency and, most importantly, they do not meet the standards of green production, which brings heavy pressure to the environment [11].

Methods involving membranes and MRs have been popular because of their green and efficient features. Membrane filtration can be used to remove large-molecule impurities in the extract, and the purification process does not cause a phase change [12]. Researchers have applied precipitation [13], chromatography [14], solvent extraction [15], and membrane methods [16] to the extraction and refinement of flavonoids. Compared with other methods, the membrane approach boasts high effectiveness, eco-friendliness, and energy conservation benefits. A macroporous adsorbent resin is created by the polymerization reaction of a monomer, crosslinking agent, porogenic agent, dispersant, and other additives. MR employment has the advantages of a high reuse rate, good safety, and low cost [17]. However, the membrane method alone is not effective in separating compounds of similar molecular weights [18]. The impurity content in the extracted solution is reduced significantly after membrane pretreatment. This phenomenon can, in terms of the MR, improve its adsorption rate and desorption rate, increase the number of times it can be used, and lengthen its service life [19]. Increasing numbers of researchers are applying membrane filtration and MR-based methods jointly to isolate and purify the efficacious substances. Researchers have applied membrane–MR coupling technology for the purification of TFs of ginkgo [20], safflower yellow pigment [21], oligofructose [18], saponin of *Cornus officinalis* [19], and phycocyanin of purple yam [12].

For the purification and separation methods of TCM formulations, the process reliability and stability of each batch must be ensured. “Fingerprinting” of TCM formulations can reflect the overall chemical composition of herbs and their relative proportions objectively. It can also characterize the “wholeness” and complexity of the material base and constituent chemical groups [22,23] in a systematic manner [24,25]. Liu et al. [26] conducted a quality control (QC) study on an extract of licorice pomace using fingerprinting. They established a QC method that was accurate, reliable, and proprietary, with stable and reproducible results. Luo and colleagues [27] purified and enriched two active ingredients in sorghum root using a MR and applied fingerprinting to establish a stable and effective QC method for the enriched material. To investigate the stability of the active ingredients in licorice residue during enrichment, fingerprinting can be used to conduct a QC study on a licorice pomace-enriched product to ensure that the enrichment process is effective and feasible.

**Figure 1:**
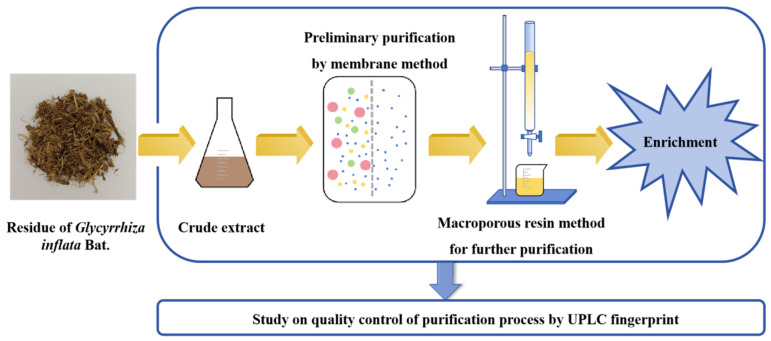
Study design.

In this study (Figure 1), a procedure was devised utilizing membrane–MR coupling technology as its foundation to prepare a high content of TFs and licochalcone A from the crude extracts of *G. inflata* Bat. We created a green and non-polluting process with high-value utilization. A QC study of the enrichment process was carried out using fingerprinting. A stable and reliable QC method was established to ensure that the flavonoid products were of stable quality and could be applied to continuous production in the industry.

## 2. Results and Discussion

### 2.1. Purification of TFs from Licorice Residue by a Membrane Method

**Figure 2:**
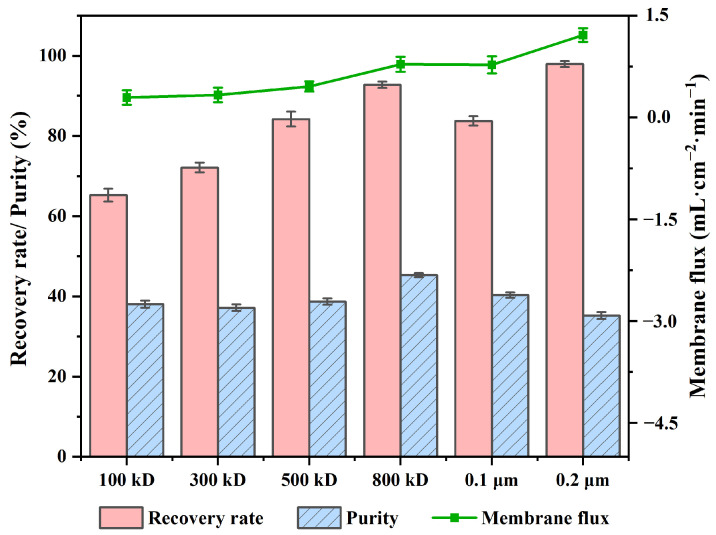
Influence of the pore size of a membrane on the recovery and purity of TFs, and membrane flux. The data represent the average ± standard deviation of three replicates.

To select the appropriate size of the membrane pore, the crude extract solution was passed through membrane sheets of pore sizes of 100 kD, 300 kD, 500 kD, 800 kD, 0.1 μm, or 0.2 μm, respectively (Figure 2). The crude extract of the licorice residue treated by the membrane with a pore size of 800 kD showed high recovery (92.77%) and purity (45.27%) of TFs. The membrane of pore size of 0.2 μm had the highest recovery and flux, but it had little effect on the purity. To achieve a better purification effect, a membrane sheet of 800 kD was selected for the next experiment.

### 2.2. MR Screening

**Figure 3:**
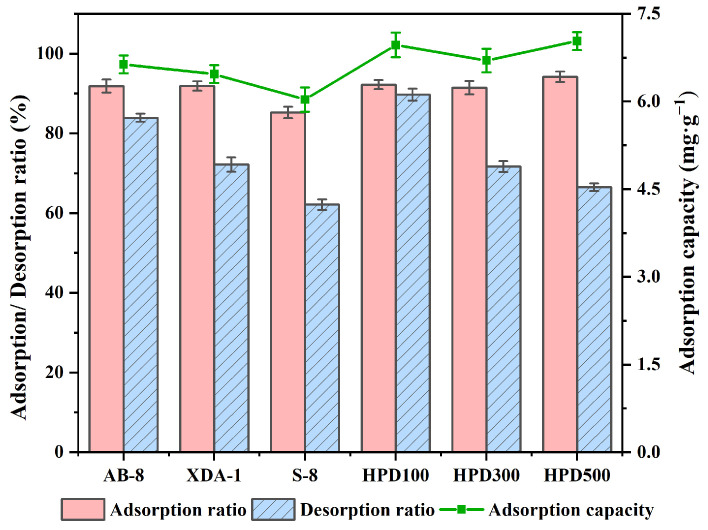
The adsorption ratio, desorption ratio, and adsorption capacity of total flavonoids on different MRs. The data represent the average ± standard deviation of three replicates.

To choose the most appropriate MR for enriching TFs, the adsorption rate, desorption rate, and adsorption capacity of TFs in six MRs were compared. The basic physical properties of six MRs are summarized in Table 1, and the findings are displayed in Figure 3. HPD-100 and HPD-500 showed excellent adsorption performance (Figure 3). The non-polar MRs. XDA-1 and HPD-300, as well as weakly polar MR AB-8, also had high adsorption capacity, but the polar MR S-8 had significantly lower adsorption and desorption capacity than the other MRs. HPD-100 demonstrated the highest capacity for desorption. The adsorption capacity of HPD-500 was marginally greater than that of HPD-100, but it had a lower desorption capacity. HPD-100 was chosen for further experiments.

### 2.3. Adsorption Kinetics of TFs from Licorice Residue on the MR HPD-100

**Figure 4:**
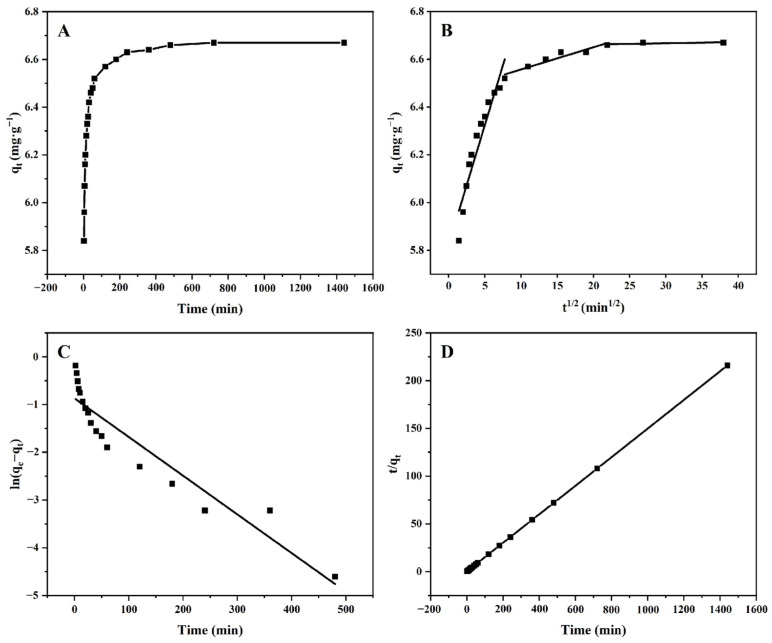
The adsorption kinetic curve (**A**) data were fitted by particle diffusion (**B**), pseudo-first-order (**C**), and pseudo-second-order (**D**) models.

Figure 4 illustrates the adsorption kinetic curve of HPD-100 for TFs. Figure 4A showed that the adsorption capacity of HPD-100 increased rapidly at 0–60 min, and the adsorption capacity nearly reached its peak at 240 min and no longer increased significantly, indicating that the adsorption reached equilibrium. This phenomenon occurred because, over time, the surface binding sites of the MR became increasingly saturated with TFs. After that, regardless of the increase in time, the adsorption capacity of the MR remained unchanged. Therefore, an adsorption time of 240 min was chosen for the next adsorption experiment.

The data for adsorption kinetics were fitted with the pseudo-first-order kinetic model, pseudo-second-order kinetic model, and intra-particle diffusion models to explain the adsorption mechanism of TFs on the MR HPD-100. Table 2 shows that the data for adsorption kinetics closely match the pseudo-second-order kinetic model, and the *R*^2^ value was 0.9999. Meanwhile, the *R*^2^ values of the pseudo-first-order and intra-particle diffusion models were 0.8817 and 0.5425–0.9173, respectively. These data showed that the adsorption process was not suitable for the pseudo-first-order kinetic model or the particle diffusion model. The theoretical adsorption capacity obtained by the pseudo-second-order kinetic model was also the closest to the actual adsorption capacity. Hence, the pseudo-second-order kinetic equation was better suited for explaining the adsorption mechanism of TFs on HPD-100.

### 2.4. Adsorption Isotherms of TFs from Licorice Residue on the MR HPD-100

**Figure 5:**
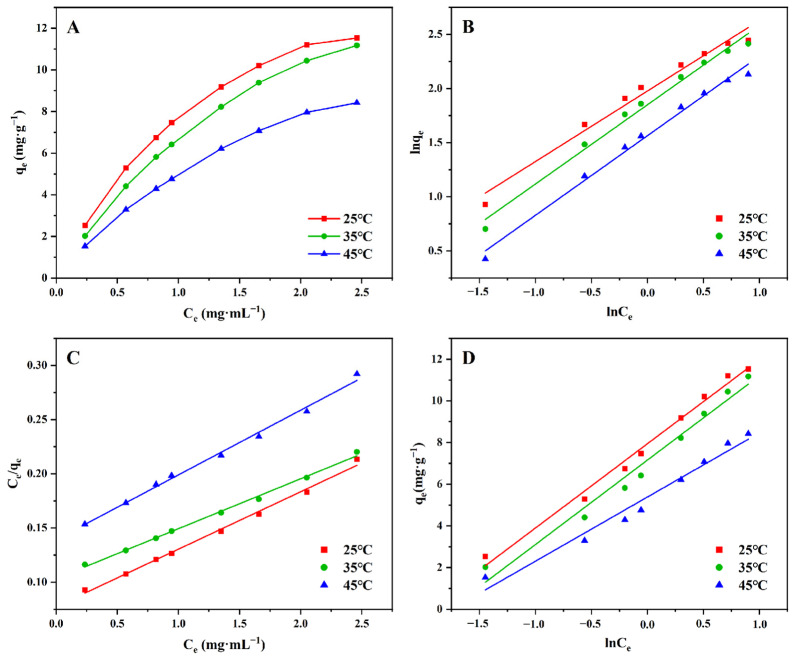
Freundlich (**B**), Langmuir (**C**), and Temkin (**D**) adsorption isotherm models were used to fit the data for the adsorption isotherm (**A**) model.

An adsorption isotherm is a curve that responds to the correlation between the adsorbent and solute in solution, and it explains the adsorption process and adsorption mechanism [28,29]. According to the method stated in Section 3.7.3, we drew the adsorption isotherm of TFs on HPD-100 (Figure 5). The adsorption capacity of HPD-100 increased rapidly at a low concentration of TFs and approached saturation gradually with an increase in concentration [30]. At the same concentration of TFs, the adsorption amount on HPD-100 decreased gradually as the temperature rose, denoting that the adsorption occurred exothermically [17].

The data for adsorption isotherms were fitted with Freundlich, Langmuir, and Temkin adsorption isotherm models, respectively. The adsorption process of TFs on the MR HPD-100 was best fitted to the Langmuir adsorption isotherm model with the highest regression coefficients (*R*^2^ = 0.993–0.996). The 1/*n* value in the Freundlich adsorption isotherm model was between 0.1 and 0.5, representing that adsorption occurred readily [31,32]. The 1/*n* values in Table 3 were > 0.5, indicating that the adsorption process was not suitable for fitting with the Freundlich model (i.e., the adsorption process was monolayer adsorption on a uniform surface) [33,34]. The average correlation coefficients of the Freundlich and Tempkin isothermal models were lower compared with that of the Langmuir isothermal model, so the Langmuir isothermal model might be a better fit for the adsorption process [35]. The larger the *K_L_* value in the Langmuir adsorption isothermal equation, the stronger the adsorption behavior [36]. The fitting results in Table 3 showed that *K_L_* decreased from 0.684 mL·mg^−1^ to 0.429 mL·mg^−1^ as temperature rose, showing that an increase in temperature reduced the adhesion of TFs. Therefore, 25 °C was determined to be the optimal temperature for adsorption.

### 2.5. Optimization of Experimental Conditions for Dynamic Adsorption

#### 2.5.1. Determination of Sample Loading Concentration

The loading concentrations studied in this experiment were 1.10, 1.72, 2.34, 3.22, 4.30, 5.41, and 6.67 mg·mL^−1^ (equivalent to 0.50, 0.78, 1.06, 1.46, 1.95, 2.45, and 3.02 mg·mL^−1^ of TFs, respectively). The adsorption capacity of the HPD-100 MR column escalated swiftly if the loading concentration was low. If the loading concentration reached 4.30 mg·mL^−1^ (equivalent to 1.95 mg·mL^−1^ of TFs), the adsorption capacity began to increase slowly, which showed that the adsorption capacity had reached saturation gradually. Selecting the appropriate loading concentration was crucial to achieve the expected purification effect. If the loading concentration is too low, the MR is not fully utilized, and the efficiency of separation and purification is low, but an excessive loading concentration will cause waste of the loading solution. Therefore, 4.30 mg·mL^−1^ was chosen as the appropriate loading concentration in this experiment.

#### 2.5.2. Determination of the Dynamic Leakage Curve

**Figure 6:**
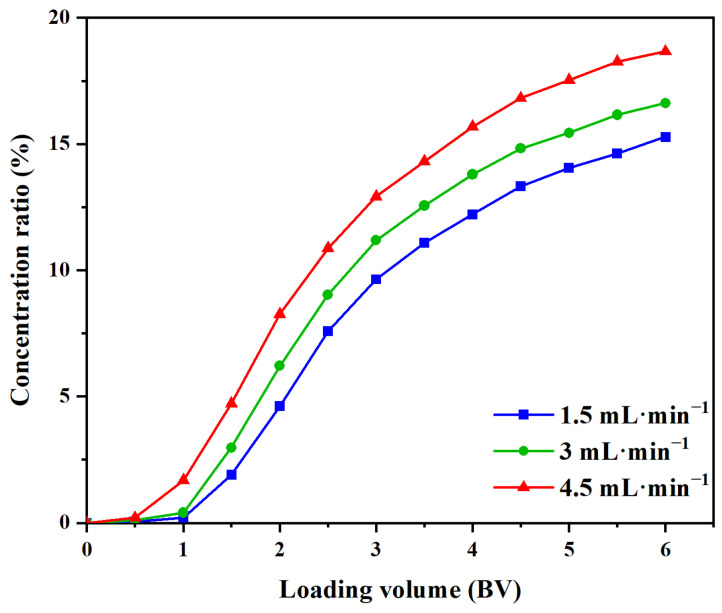
Leakage curves of TFs on the MR HPD-100 at different loading speeds.

We wished to determine the optimal flow rate and leakage point. Hence, the leakage curves for different sample loading velocities were plotted. The arrival of the leakage point means that the MR has reached absorption saturation, causing the sample solution to leak from between the resin particles. This, in turn, resulted in a wastage of the sample solution and reduced purification efficiency. In general, it is accepted that the leakage point is reached if the effluent concentration reaches 10% of the loading solution concentration [37]. The best sample loading volume and flow rate can be determined by judging the leakage point. The TF concentration in the effluent increased with an increase in sample loading (Figure 6). When the loading volume was 2.5 BV, and the loading solution concentration was 4.30 mg·mL^−1^ (equivalent to 1.95 mg·mL^−1^ of TFs), the leakage point occurred prematurely with a sample loading speed of 4.5 mL·min^−1^. Furthermore, when the sample volume was 2.5 BV, the leakage point of the curve with a sample loading speed of 4.5 mL·min^−1^ was earlier than that with a loading speed of 3 mL·min^−1^. At 3 BV, the leakage point with the sample loading speed of 3 mL·min^−1^ was also reached. To save time and ensure the purification effect, the sample loading volume was thus determined to be 2.5 BV, and the sample loading speed was 3 mL·min^−1^.

### 2.6. Determination of Optimal Application Sequence of Membrane Method and MR Method

The optimal application sequence of the membrane method and MR method in the process of combination was investigated by comparing the purity of TFs and licorice chalcone A in flavonoid products prepared using both purification methods. The findings are displayed in Table 4. As evident from the table, method ② (the membrane method followed by the MR method) showed the best purification of the unrefined licorice residue extract, and the TF purity and licochalcone, A content of the flavonoid products produced were higher than those of method ①. This may be due to the fact that most of the impurities were removed from the sample purified by the MR in method ①, which resulted in a more homogeneous molecular weight. Therefore, continuing to use the membrane method for purification did not have a significant effect, and the desired purification was not achieved. In method ②, the membrane method significantly reduced most large molecular weight impurities in the crude extract of the licorice residue, which transformed the liquid from viscous and turbid to clear and transparent, thereby improving the adsorption rate of the pore of the macroporous adsorbent resin for the effective substances, enhancing the purification performance of the MR, and significantly improving the purification effect. Therefore, the optimal order of the combined methods is to use the membrane method for primary purification and then use MR for further purification.

### 2.7. Optimization of the Desorption Conditions of the MR HPD-100

RSM was used to optimize the desorption parameters of TFs by HPD-100. Different desorption conditions will simultaneously affect the recovery rate and purity of TFs in licorice, and a high recovery rate does not necessarily mean high purity. Therefore, finding a balance point between the two to achieve maximum enrichment was crucial for this experiment. The selected variables and response values can be found in Table 5. The results of 17 groups of tests are presented in Table 6.

The high F value of the model (Table 7) indicated that the model was highly significant. The *p*-value of the regression model reached significance (*p* < 0.05), and the lack of fit was insignificant (*p* > 0.05). These data showed the established model was well-fitted, the regression was significant and reliable, and the model was established [38,39]. The predicted *R*^2^ and adjusted *R*^2^ in the two models were reasonably consistent, and the accuracy ratio was 12.907 and 21.715, respectively, indicating that the model could assist in exploring the design parameters. In the elution process, the importance of each factor was B > A > C, indicating that the eluent concentration was the most critical factor, which had a great influence on the recovery and purity of TFs, whereas the eluent flow rate was not important in this process.

The results in Table 7 were analyzed by fitting a quadratic equation with Design Expert software v.10.0. The quadratic multiple regression equations of the recovery and purity of TFs were obtained: recovery rate (%) = 90.84 + 1.01A + 5.69B + 0.60C − 0.37AB − 0.050AC − 0.15BC − 2.58A^2^ − 10.33B^2^ − 1.06C^2^; purity (%) = 77.70 − 2.03A + 2.36B + 0.24C − 1.98AB + 0.12AC + 0.100BC − 4.20A^2^ − 6.48B^2^ − 2.82C^2^.

**Figure 7:**
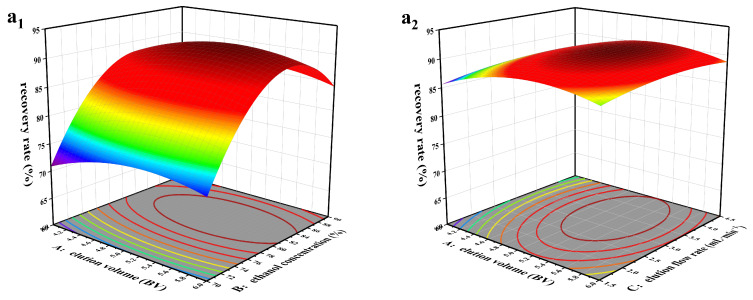

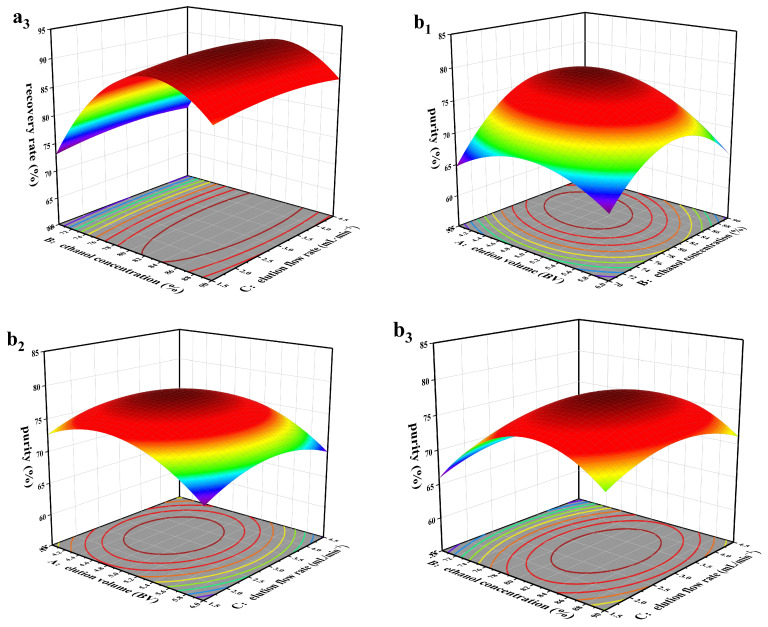
Response surface graph of variables to the recovery rate (**a_1_**–**a_3_**) and purity (**b_1_**–**b_3_**) of total flavonoids. (**a_1_**,**b_1_**) Interaction of elution volume and ethanol concentration. (**a_2_**,**b_2_**) Interaction of elution volume and elution flow rate. (**a_3_**,**b_3_**) Interaction of ethanol concentration and elution flow rate.

Figure 7 shows the influence of the interaction of three variables on individual recovery and purity. The predicted optimal conditions were obtained: elution volume = 4.829 BV, ethanol concentration = 82.429%, elution flow rate = 3.132 mL·min^−1^.

### 2.8. Validation Experiment

In practice, the MR HPD-100 was eluted with an elution volume of 5 BV, the ethanol concentration was 83%, and the elution speed was 3 mL·min^−1^. In these cases, the recovery and purity of TFs were 91.44% and 78.2%, respectively, the content of licochalcone A was 22.70 mg·g^−1^, and all of the RSD values were <2%.

### 2.9. Establishment of a UPLC Fingerprint

**Figure 8:**
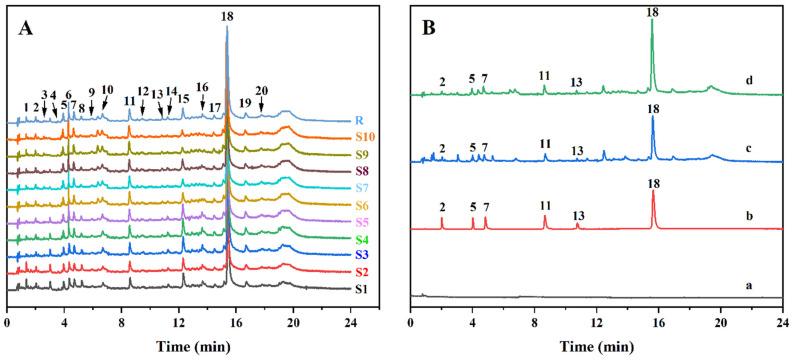
(**A**): UPLC fingerprint of 10 batches of licorice residue-enriched substances and control chromatogram (R). (**B**): Comparison plots of the enriched material with the crude extract, mixed standard, and blank solvent. a: blank solvent; b: mixed standard; c: crude extract; d: enriched material; 2: liquiritin; 5: isoliquiritin; 7: liquiritigenin; 11: isoliquiritigenin; 13: ammonium glycyrrhizinate; 18: licochalcone A.

The fingerprint of TFs-enriched substances in *G. inflata* Bat. residue was established by UPLC. The chromatogram S1 was used as the reference chromatogram, and the average method was used to generate the control chromatogram (R) (Figure 8A). Twenty common peaks were extracted from 10 batches of enrichment, and similarity matching was >0.98. Hence, the established fingerprints could be used for the QC of the TFs-enriched substances from licorice pomace. Comparison with the chromatogram of the mixed reference substance enabled six flavonoid components to be identified from the TFs-enrichment of licorice residue: liquiritin, isoliquiritin, liquiritigenin, isoliquiritigenin, ammonium glycyrrhizinate, and licochalcone A (Figure 8B). The content of all six TF components increased after purification of the crude extract of licorice pomace, among which the content of licochalcone A increased the most, from 11.63 mg·g^−1^ to 22.70 mg·g^−1^ (1.95-fold), suggesting that this process was also suitable for the enrichment of licochalcone A.

## 3. Materials and Methods

### 3.1. Raw Materials and Other Materials

The raw material of *G. inflata* Bat. residue was from Xinjiang Kunlun Shennong (Kashi, China) and was kept at room temperature. Macroporous adsorbent resins (AB-8, XDA-1, S-8, HPD-100, HPD-300, HPD-500) were acquired from Zhengzhou Hecheng New Material Technology (Zhengzhou, China). Polyvinylidene-fluoride membranes (100 kD, 300 kD, 500 kD, 800 kD, 0.1 μm, 0.2 μm) were supplied by Guxin Biotechnology (Shanghai, China).

### 3.2. Reagents and Instruments

Anhydrous ethanol was acquired from Tianjin Fuyu Fine Chemicals (Tianjin, China). Formic acid was obtained from Tianjin Yongsheng Fine Chemicals (Tianjin, China). Hydrochloric acid was sourced from Chengdu Cologne Chemicals (Chengdu, China). Sodium hydroxide was bought from Tianjin Yongsheng Fine Chemicals (Tianjin, China). Chromatography-grade acetonitrile was from Thermo Fisher Scientific (Waltham, MA, USA). Potassium hydroxide was bought from Tianjin Sheng’ao Chemical Reagents (Tianjin, China).

An ultraviolet (UV) spectrophotometer (UV-2600) was purchased from Shimadzu (Kyoto, Japan). An H-Class ultrahigh pressure liquid chromatography (UPLC) system (Acquity) was obtained from Waters (Milford, MA, USA). A tangential-flow nanofiltration membrane system was sourced from Guxin Biotechnology (Shanghai, China). A constant-temperature oscillator (SHZ-82) was acquired from Jinyi Instrument Technology (Jiangsu, China). A rotary evaporator (RE-2000A) was sourced from Yarong Biochemical Instruments (Shanghai, China). The circulating water-type multi-purpose vacuum pump (SHZ-D) was obtained from Kerui Instruments (Gongyi, China). An ultrasonic cleaner (KQ-500DE CNC) was purchased from Ultrasonic Instruments (Kunshan, China). A vacuum drying oven (DZF-6050) was obtained from Xinmiao Medical Equipment Manufacturing (Shanghai, China).

### 3.3. Preparation of an Extract of Licorice Residues

We removed debris (e.g., earth, stone, plastic) from the licorice residue and removed floating soil and fine sand with a 40-mesh sieve. Dry licorice residues (120 g) were refluxed and extracted with 90% ethanol (*v*/*v*) for 1 h twice, and the material ratio was 1:5 and 1:3, respectively. After that, the culture was gathered, and crude extracts were enriched by rotary evaporation at a reduced pressure of 60 °C. Vacuum drying was conducted for 2 h at 50 °C, and the material was removed for later use.

### 3.4. Determination of TFs and Licochalcone A in Licorice Residue

#### 3.4.1. Determination of TF Content by UV Spectrophotometry

Using liquiritin separated from TFs as standard, the content of TFs was determined by UV spectrophotometry [40]. We placed the solution to be tested (0.2 mL) in a 10-mL volumetric flask and mixed it with 0.5 mL of potassium hydroxide solution (10%). After 5 min, record the absorbance intensity at 335 nm (Supplemental Figure S1) with a spectrophotometer (UV-2600; Shimadzu, Kyoto, Japan). The standard curve of liquidity was given by *A* = 0.0589*C* + 0.0128 (*R*^2^ = 0.9990, 3.72–13.02 μg·mL^−1^).

The recovery rate of TFs could be given by Equation (1):

(1) $$R(\%)=\frac{C_{2}\times V_{2}}{C_{1}\times V_{1}}\times 100\%$$

here *C*_1_ (mg·mL^−1^) represents the concentration and *V*_1_ (mL) stands for the volume of a TF solution before treatment, and *C*_2_ (mg·mL^−1^) represents the concentration, and *V*_2_ (mL) stands for the volume of a TF solution after treatment, respectively.

The purity of TFs can be given by Equation (2):

(2) $$P(\%)=\frac{C\times V}{M}\times 100\%$$

here *C* (mg·mL^−1^) stands the concentration, *V* (mL) indicates the volume of TFs in a solution, and *M* (mg) is the sample’s weight.

#### 3.4.2. Determination of Licochalcone A by UPLC

A waters ACQUITY H-Class Series UPLC system consisting of a quaternary solvent manager, a sample manager, and a photo-diode array detector was used for UPLC-PAD analysis. Samples were gradient eluted through a Unitary C_18_ column with a column temperature of 30 °C. The volume injected was 1 microliter with (A) acetonitrile and (B) formic acid (0.2%) in gradient elution mode (0–2 min, 25% A; 2–10 min, 40% A; 10–17 min, 50% A; 17–18 min, 60% A; 18–20 min, 20% A; 20–24 min, 20% A). The flow rate was 0.3 mL/min^−1^, and the detection wavelength was 372 nm. The standard curve of licochalcone A was as follows: *A* = 14539C + 9514.6 (*R*^2^ = 0.9998, 8.00–80.00 μg·mL^−1^).

### 3.5. Purification by a Membrane Method

#### 3.5.1. Screening of the Pore Size of A Membrane

The tangential-flow nanofiltration membrane system (GS-NF400) was used for membrane filtration of an extract of licorice residue. The pore size of the membrane was 100 kD, 300 kD, 500 kD, 800 kD, 0.1 or 0.2 μm, and the surface area of the membrane was 216 cm^2^. The transmembrane pressure (TMP) during membrane filtration was 0.1 MPa, pump speed frequency (Q_f_) of 20 Hz, and temperature of 35 °C. The inlet and outlet ends were placed in a liquid storage tank, and the solution at the filter end was collected simultaneously. After the start of filtration, the amount of liquid in the inlet storage tank gradually decreased. When the amount of liquid in the inlet storage tank became too low, the membrane filtration system could no longer operate normally. Therefore, it became necessary to add more solvent and continue filtration. Filtration was stopped after the solvent had been replenished five times [18,21].

The membrane flux (*J_w_*) and recovery rate of TFs (*R*) were used to evaluate the performance of the membrane.

The membrane flux was tracked by measuring the volume of filtrate collected at a set interval with a graduated cylinder in accordance with Equation (3) [41]:

(3) $$J_{W}=\frac{V}{t\times S}$$

where *J_w_* (mL·cm^−2^·min^−1^) is the membrane flux, *V* (mL) is the permeate volume, *t* (min) is the duration, and *S* (cm^−2^) is the effective membrane area.

#### 3.5.2. Cleaning and Maintenance of the Membrane

The original permeability of the membrane was restored using a cleaning-in-place procedure [42]. Rinse the membrane with pure water for 20 min to remove impurities adhering to the membrane surface. Then, it was washed with 70% ethanol for 20 min to remove alcohol-soluble impurities. Next, it underwent a 20-min rinse with a hydroxide solution (0.2 mol·L^−1^). The membrane was removed and stored in sodium hydroxide solution (0.1 mol·L^−1^). The washing operation was carried out at a TMP of 0.1 MPa, Q_f_ of 20 Hz, and with a mean temperature of 45 °C.

### 3.6. MR Pretreatment

The MR was immersed in absolute ethanol for 12 h, washed with distilled water, and then immersed in 5% sodium hydroxide (*m*/*v*) and HCL (*v*/*v*) solution in turn. Next, rinse the MR with distilled water until it is neutral. Then, the MR was immersed in absolute ethanol and subsequently rinsed with water again until there was no trace of ethanol.

### 3.7. Tests of Static Adsorption and Desorption

#### 3.7.1. MR Screening

When selecting a suitable MR, we mainly choose the adsorption rate, adsorption capacity, and desorption rate as evaluation indicators. We weighed six types of 2.0 g-pretreated MRs into a 100-mL conical flask and accurately added 15 mL of TF solution (1.0 mg·mL^−1^). Next, the flask was positioned within a temperature-controlled shaker at 25 °C for 24 h (120 rpm). After spinning down the supernatant, the MR was rinsed twice with distilled water. The washed MR was shaken in 50 mL of 80% (*v*/*v*) ethanol for 24 h (25 °C, 120 rpm). Measured the absorbance after adsorption and desorption and calculated the concentration of TF. Repeated each treatment thrice. The formulas used to calculate the adsorption ratio, absorption capacity, and desorption ratio of various MRs are shown in Equations (4), (5) and (6), respectively.

Adsorption ratio:

(4) $$E(\%)=\frac{C_{0}-C_{1}}{C_{0}}\times 100\%$$

Absorption capacity:

(5) $$Q(\%)=\frac{(C_{0}-C_{1})\times V_{1}}{W}\times 100\%$$

Desorption ratio:

(6) $$P(\%)=\frac{C_{2}\times V_{2}}{(C_{0}-C_{2})\times V_{1}}\times 100\%$$

where *C*_0_ (mg·mL^−1^) stands the initial TF concentration prior to adsorption, *C*_1_, *C*_2_ (mg·mL^−1^) denotes the TF concentration in the sample solution post-adsorption and after absorption, severally, and *V*_1_ (mL) and *V*_2_ (mL) denote the volumes of sample and desorption solution, severally. *W* (g) represents the dry MR’s weight [3,17].

#### 3.7.2. Adsorption Kinetics of TFs on the MR HPD-100

The pretreated HPD-100 MR was weighed accurately (2.0 g) and kept in a 100-mL conical flask. Next, 15 mL of a TF solution (0.944 mg·mL^−1^) was added and then placed in a thermostatic oscillator for adsorption. We removed 1 mL of solution at 2, 4, 6, 8, 10, 15, 20, 25, 30, 30, 40, 50, 60, 90, 120, 180, 240, 360, 480, 720, and 1440 min. The TF content was measured, and the adsorption kinetic equation was established. A pseudo-first-order kinetics model, pseudo-second-order kinetics model, and intra-particle diffusion model were employed to model the adsorption kinetic data, as shown by Equations (7), (8) and (9), respectively [17,43,44].

Pseudo-first-order kinetic equation:

(7) $$\ln(q_{e}-q_{t})=\ln q_{e}-K_{1}t$$

Pseudo-second-order kinetic equation:

(8) $$\frac{t}{q_{t}}=\frac{1}{{K_{2}q_{e}}^{2}}+\frac{t}{q_{e}}$$

Intra-particle diffusion model:

(9) $$q_{t}=K_{d}t^{\frac{1}{2}}+C$$

*K*_1_, *K*_2_, and *K_d_* represented the rate constants for pseudo-first-order, pseudo-second-order, and particle diffusion adsorption kinetic, respectively. *C* was a constant in the kinetic model of segmental migration.

#### 3.7.3. Adsorption Isotherms of TFs on the MR HPD-100

We accurately weighed three parts of the HPD-100 MR (2.0 g) and transferred them to 100-mL conical flasks, respectively. Then, we added 15 mL of a TF solution to them and placed them in a constant-temperature oscillator at 25 °C, 35 °C, or 45 °C for adsorption. After shaking at different temperatures for 240 min, centrifugation was carried out to determine the TF content in the supernatant, and the adsorption isotherms at different temperatures were obtained. The adsorption isotherm can determine the adsorption capacity, saturation time, adsorption rate, and other important parameters of the adsorbent, which can explain the adsorption mechanism and adsorption properties of the adsorbent. The Freundlich adsorption model (Equation (10)), Langmuir adsorption model (Equation (11)), and Temkin adsorption model (Equation (12)) were utilized to simulate the adsorption isotherms. Then, the adsorption properties of TFs on HPD-100 were described.

Freundlich adsorption model [45]:

(10) $$\ln q_{e}=\frac{\ln C_{e}}{n}+\ln K_{F}$$

Langmuir adsorption model [46]:

(11) $$\frac{C_{e}}{q_{e}}=\frac{1}{K_{L}q_{m}}+\frac{C_{e}}{q_{m}}$$

Temkin adsorption model [47,48]:

(12) $$q_{e}=B_{T}\ln K_{T}+B_{T}\ln C_{e}$$

*K_F_* represents the adsorption coefficient for the Freundlich adsorption model, and 1/*n* reflects the adsorption intensity. *K_L_* represents the Langmuir adsorption coefficient, and *q_m_* indicates the saturated adsorption capacity. *K_T_* and *B_T_* are the adsorption constants of the Temkin adsorption model.

### 3.8. Optimization of Experimental Conditions for Dynamic Adsorption

#### 3.8.1. Determination of Sample Loading Concentration

We weighed 20.0 g of dry HPD-100, wet-mounted the column, and deposited for 1 h to make the MR column compact and bubble-free. The loading solution with concentrations of 1.10, 1.72, 2.34, 3.22, 4.30, 5.41, and 6.67 mg·mL^−1^ (equivalent to 0.50, 0.78, 1.06, 1.46, 1.95, 2.45, and 3.02 mg·mL^−1^ of TFs) was adsorbed by the MR column with a flow rate of 3 mL·min^−1^. The effluent was collected, and the TF content in the effluent was determined by UV spectrophotometry. All experiments were carried out thrice in parallel.

#### 3.8.2. Determination of the Dynamic Leakage Curve

The loading solution at 4.30 mg·mL^−1^ (equivalent to 2.0 mg·mL^−1^ of TFs) was loaded into the HPD-100 MR column (equivalent to 20 g of dry MR) at speeds of 1.5, 3.0, or 4.5 mL·min^−1^. One tube of effluent was gathered at intervals of 0.5 BV for the assessment of the TF content using UV spectrophotometry. The optimal loading volume and loading speed were determined by plotting the dynamic leakage curve, where the abscissa represents the volume of effluent, and the ordinate signifies the TF concentration in each effluent sample.

### 3.9. Investigation of Membrane and MR Method Application Order

#### 3.9.1. Effect of MR-Membrane Method on Purification

To prepare the crude extract solution, licorice residue crude extract powder was weighed and then loaded onto HPD-100 resin for adsorption under optimized conditions, followed by elution with 80% ethanol. The eluate was collected and prepared as a sample solution having a TF concentration of 1.0 mg·mL^−1^. The filter solution was prepared following the membrane and filtration procedure selected in Section 3.5.1. The contents of TFs and licochalcone A in the filter solution were then determined. All tests were carried out three times in parallel.

#### 3.9.2. Effect of the Membrane–MR Method on Purification

To create a sample solution containing TF with a concentration of 1.0 mg·mL^−1^, licorice residue crude extract powder was first weighed. Then, the filtrate was prepared with the membrane and filtration method screened in Section 3.5.1. Next, the filtrate was loaded onto HPD-100 resin for adsorption under the selected optimal conditions. The resin was subsequently eluted with 80% ethanol, and the eluent was collected, after which the content of TFs and licochalcone A were determined in the eluate. All tests were conducted three times in parallel.

### 3.10. Optimization of Desorption Conditions of the MR HPD-100

Response surface methodology (RSM) was used to refine the optimization of the parameters of TF desorption using HPD-100. The Box–Behnken design (BBD) in Design Expert v.10.0 (Stat Ease, Minneapolis, MN, USA) was employed to design the experiment. The elution conditions were optimized with elution volume, ethanol concentration, and elution flow rate as variables and the recovery and purity of TFs as response values. RSM was employed to examine the correlation between predictor variables and response variables. Multiple regression analysis was conducted using the experimental data, and the corresponding polynomial model was fitted. Analysis of variance (ANOVA) verified the effectiveness of the model, and *p* < 0.1 indicated that the model exhibited significance.

### 3.11. QC of Enriched Substances

#### 3.11.1. Preparation of Mixed Standard and Test Sample

We accurately weighed an appropriate amount of liquiritin, isoliquiritin, liquiritigenin, isoliquiritigenin, ammonium glycyrrhizinate, and licochalcone A. Next, we added 70% methanol to prepare a mixed reference substance at a certain concentration and kept it in a refrigerator at 4 °C. We accurately weighed 10 mg of the sample and diluted it with 70% methanol in a 10-mL volumetric flask. All samples required filtration with a 0.22-μm membrane.

#### 3.11.2. Simultaneous Determination of Six Components by UPLC

A UPLC system (Acquity H-Class) consisting of a quaternary solvent manager, sample manager, and a photo-diode array detector is utilized for analyses. The sample was gradient eluted through a unitary C_18_ column with a column temperature of 30 °C. The injection volume was 1 μL with (A) acetonitrile and (B) formic acid (0.2%) in gradient elution mode (0–2 min, 25% A; 2–10 min, 40% A; 10–17 min, 50% A; 17–18 min, 60% A; 18–20 min, 20% A; 20–24 min, 20% A). The flow rate was 0.3 mL·min^−1^. According to the UV-absorption characteristics of the six components (Supplemental Figure S2), the detection wavelength was set (0–3 min: 276 nm; 3–4. 3 min: 365 nm; 3–7 min: 276 nm; 7–10 min: 372 nm; 5 min: 250 nm; 11.5–24 min: 372 nm). The chromatographic peak of six components was determined via the retention time.

#### 3.11.3. Methodology Validation

The same sample was determined on six occasions to evaluate the precision. The relative standard deviation (RSD) for the relative retention time of the main chromatographic peaks was 0.04–0.5%, and the RSD of the relative peak area was 0.83–2.99%. These findings indicated that the method demonstrated high accuracy of the testing equipment. We prepared six test samples in parallel in accordance with the description in Section 3.11.1 and injected samples in accordance with the chromatographic conditions stated in Section 3.11.2 to determine their reproducibility. The RSD of the relative retention time of the main chromatographic peaks was 0.07–0.44%, and the RSD of the relative peak area was 0.75–2.15%. These findings demonstrated that the method exhibited excellent reproducibility. The identical sample was assessed at 0, 2, 4, 8, 12, and 24 h for stability study. The RSD of the relative retention time of the main chromatographic peaks was 0.07–0.52%, and the RSD of the relative peak area was 0.84–2.42%. These results indicated that the sample remained relatively stable over a period of 24 h.

#### 3.11.4. Establishment of a UPLC Fingerprint

Ten batches of TFs-enriched substances were prepared in parallel. Samples were injected in accordance with the chromatographic conditions stated in Section 3.11.2, and the chromatogram was recorded. The obtained chromatographic data were exported to Computable Document Format at the Empower™ workstation (Waters) and entered into the similarity-evaluation system of chromatographic fingerprints of TCM (2012.130723 version). The similarity analysis of 10 batches of enriched substances was carried out. The chromatogram S1 was used as the reference chromatogram, and an averaging method was used to generate the control chromatogram. The width of the time interval was adjusted to 0.1 min.

## 4. Conclusions

The crude extract of licorice residue was preliminarily enriched by a membrane method. A filter membrane sheet of size 800 kD was selected because of its high recovery and purity. The purity of TFs increased from 32.9% to 45.27%, and the recovery rate was 92.77%. At the same time, recovery and purity were used as evaluation indicators, and the optimal resin, HPD-100, was selected from six types of MRs. Additionally, the adsorption mechanism and purification conditions of TFs on the resin were investigated. Next, the optimal application sequence of the membrane and MR methods was investigated. It was found that the expected purification effect could be achieved by using a membrane for initial purification, followed by the MR method for further purification. Then, RSM was applied to optimize the desorption conditions of the membrane-purified TFs on the resin. This purification process increased TF purity to 78.2% with a recovery rate of 91.44%, and the content of licochalcone A increased from 11.63 mg·g^−1^ to 22.70 mg·g^−1^ (1.95-fold). Compared with the single purification process, the membrane–MR coupling process could overcome the drawbacks of the similar molecular weights of components and poor separation effect if the membrane method alone is used. Furthermore, the membrane method made the impurity content of the crude extract solution significantly lower and improved the adsorption rate of the MR on effective substances, which led to an obvious improvement in the purification effect. This process could replace the traditional methods of alkali dissolution and acid precipitation to save cost and reduce pollution to the environment, which provides a basis for the scaled-up application in industry.

UPLC established the fingerprints of the TF enrichment of licorice residue. The similarity of all 10 batches of enriched compounds was >0.98, which indicated that the TFs-enriched compounds of licorice residue from each batch were well correlated, and the enrichment process was stable and reliable. The main monomer compounds were analyzed as the QC components to furnish a (i) reference for the development and utilization of *G. glabra* pomace and the QC of its flavonoid products and (ii) a basis for the further rational use of *G. glabra* resources.

## Figures and Tables

**Table 1:** Basic physical properties of six MRs made of polystyrene.

MR	Particle Size (mm)	Specific Surface Area (m^2^·g^−1^)	Average Pore Diameter (Å)	Polarity
HPD-100	0.3–1.25	500–550	85–90	Non
AB-8	0.3–1.25	480–520	130–140	Weak
HPD-300	0.3–1.25	800–870	50–55	Non
HPD-500	0.3–1.25	500–550	100–120	Non
XDA-1	0.3–1.25	1000–1100	85–95	Non
S-8	0.3–1.25	100–120	280–300	Polarity

**Table 2:** Dynamic parameters of pseudo-first-order, pseudo-second-order, and particle diffusion models.

Dynamics Model	Kinetic Equation	Parameters
Pseudo-first order model	ln(*q_e_* − *q_t_*) = ln*q_e_* − *K*_1_*t*	*K*_1_ = 0.0081 min
		*q_e_* = 0.419 mg·g^−1^
		*R*^2^ = 0.88173
Pseudo-second order model	*t*/*qt* = 1/*K*_2_*q_e_*^2^ + *t*/*q_e_*	*K*_2_ = 0.1304
		*q_e_* = 6.674
		*R*^2^ = 0.999997
Intra-particle diffusion model	*q_t_* = *K_d_t*^1/2^ + *C*	*K_d_*_1_ = 0.1004
		*C*_1_ = 5.823
		*R*^2^ = 0.9087
		*K_d_*_2_ = 0.00929
		*C*_1_ = 6.456
		*R*^2^ = 0.9173
		*K*_d3_ = 5.176 × 10^−4^
		*C*_1_ = 6.65
		*R*^2^ = 0.5425

**Table 3:** Parameters for the Freundlich, Langmuir, and Temkin adsorption isotherms.

Model	T/(°C)	Equations	Parameters		
			*K_F_* [(mg·g^−1^) (mL·mg^−1^) ^1/*n*^]	*n*	*R* ^2^
Freundlich	25	ln*q_e_* = 0.651 ln*C_e_* + 1.977	7.220	1.535	0.974
	35	ln*q_e_* = 0.732 ln*C_e_* + 1.850	6.359	1.367	0.986
	45	ln*q_e_* = 0.735 ln*C_e_* + 1.564	4.778	1.361	0.988
			*K_L_* (mL·mg^−1^)	*q_m_* (mg·g^−1)^	*R* ^2^
Langmuir	25	$\frac{Ce}{qe}\;$= 0.0530*C_e_* + 0.0775	0.684	18.875	0.994
	35	$\frac{Ce}{qe}$ = 0.0460*C_e_* + 0.103	0.446	21.715	0.996
	45	$\frac{Ce}{qe}\;$= 0.0597*C_e_* + 0.139	0.429	16.742	0.993
			*K_T_* (L·mg^−1^)	*B_T_* (J·mol^−1^)	*R* ^2^
Temkin	25	*q_e_* = 4.041 ln*C_e_* + 7.942	7.137	4.041	0.988
	35	*q_e_* = 4.048 ln*C_e_* + 7.157	5.859	4.048	0.973
	45	*q_e_* = 3.077 ln*C_e_* + 5.386	5.756	3.077	0.967

**Table 4:** Purity of total flavonoids, licochalcone A content, and their changes under different purification sequences.

Serial Number	Application Sequence	Purity of Total Flavonoid (%)	Change in Purity (Multiplication Factor)	Content of Licochalcone A (mg·g^−1^)	Change in Content (Multiplication Factor)
①	MR–membrane method	47.6 ± 1.26	1.45	15.23 ± 0.53	0.31
②	Membrane–MR method	68.7 ± 1.23	2.09	19.94 ± 1.13	1.71

**Table 5:** Independent factors and levels of the elution conditions of the MR HPD-100.

Independent Factor	Level		
	−1	0	1
Elution volume	4	5	6
Ethanol concentration	70	80	90
Elution flow rate	1.5	3	4.5

**Table 6:** Box–Behnken experimental design and results.

run	A (BV)	B (%)	C (mL·min^−1^)	Recovery Rate (%)	Purity (%)
1	5	70	1.5	72.3	66
2	6	80	4.5	87.3	68.7
3	6	70	3	74	65.2
4	5	90	1.5	83.6	70.7
5	5	80	3	93.3	78.6
6	5	70	4.5	75.6	65.9
7	5	80	3	92.8	78.3
8	5	80	3	88.4	77
9	4	80	1.5	87	72.9
10	4	80	4.5	86.5	73.5
11	4	70	3	70.1	64.3
12	5	80	3	89	77.3
13	5	80	3	90.7	77.3
14	6	80	1.5	88	67.6
15	5	90	4.5	86.3	71
16	6	90	3	85	65.8
17	4	90	3	82.6	72.8

**Table 7:** ANOVA for the response surface regression model.

RECOVERY RATE							Purity					
*R* ^2^	*R*^2^ = 0.9645		*R*_A_^2^ = 0.9188		*R*_P_^2^ = 0.7774		*R*^2^ = 0.9895		*R*_A_^2^ = 0.9760		*R*_P_^2^ = 0.9013	
Adequate precision	12.907						21.715					
Source	SS	df	MS	F-Value		*p*-value	SS	df	MS	F-Value		*p*-value
Model	774.65	9	86.07	21.11		0.0003	406.68	9	45.19	73.18		<0.0001
A (elution volume)	8.20	1	8.20	2.01		0.1991	32.80	1	32.80	53.13		0.0002
B (ethanol concentration)	258.78	1	258.78	63.46		<0.0001	44.65	1	44.65	72.31		<0.0001
C (elution flow rate)	2.88	1	2.88	0.71		0.4285	0.45	1	0.45	0.73		0.4209
AB	0.56	1	0.56	0.14		0.7213	15.60	1	15.60	25.27		0.0015
AC	1 × 10^−2^	1	1 × 10^−2^	2.452 × 10^−3^		0.9619	0.063	1	0.063	0.10		0.7597
BC	0.090	1	0.090	0.022		0.8861	0.040	1	0.040	0.065		0.8064
A^2^	28.08	1	28.08	6.89		0.0342	74.27	1	74.27	120.28		<0.0001
B^2^	449.52	1	449.52	110.24		<0.0001	176.53	1	176.53	285.88		<0.0001
C^2^	4.71	1	4.71	1.15		0.3182	33.60	1	33.60	54.42		0.0002
Residual	28.54	7	4.08				4.32	7	0.63			
Lack of fit	9.29	3	3.10	0.64		0.6263	2.34	3	0.78	1.58		0.3269
Pure error	19.25	4	4.81				1.98	4	0.50			
Correlation total	803.20	16					411.00	16				

## Data Availability

Data are contained within the article.

## Supplementary Materials

The following supporting information can be downloaded at: https://www.mdpi.com/article/10.3390/molecules29102282/s1, Figure S1: UV spectrum of TFs in licorice residue; Figure S2: UV-vis spectra of six components (1: liquiritin; 2: isoliquiritin; 3: liquiritigenin; 4: isoliquiritigenin; 5: ammonium glycyrrhizinate; 6: licochalcone A).
