# Supplementary material for: Enrichment of Total Flavonoids and Licochalcone A from Glycyrrhiza inflata Bat. Residue Based on a Combined Membrane–Macroporous Resin Process and a Quality-Control Study
Source: Molecules. 2024 May 12;29(10):2282. doi: 10.3390/molecules29102282 (PMC11124024; doi:10.3390/molecules29102282)
Supplement: Supplementary file 1 [file molecules-29-02282-s001.zip › molecules-2979099-supplementary.pdf]

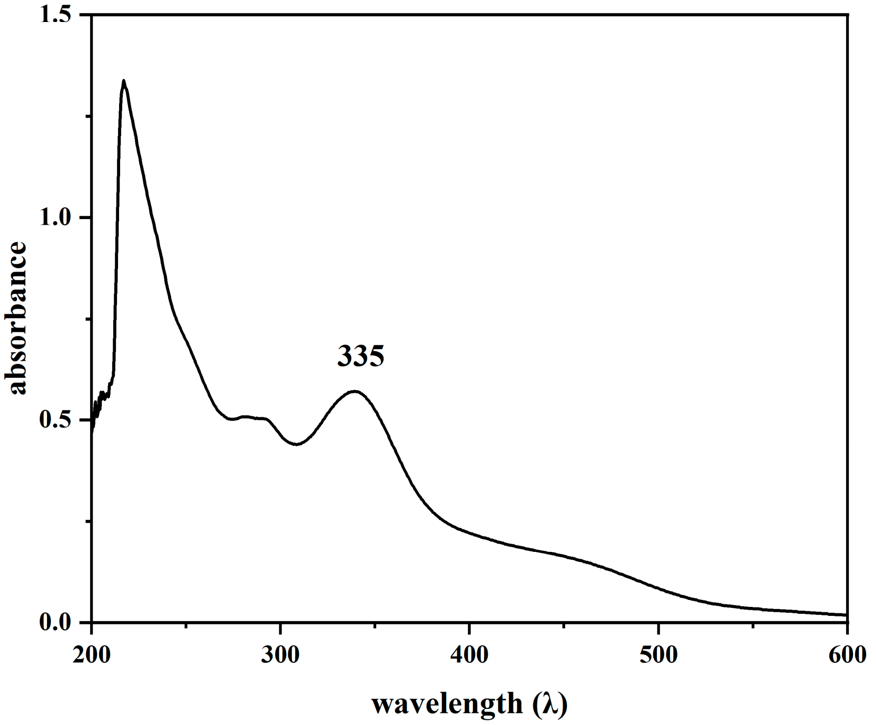

Supplemental Figure S1 UV spectrum of TFs in licorice residue

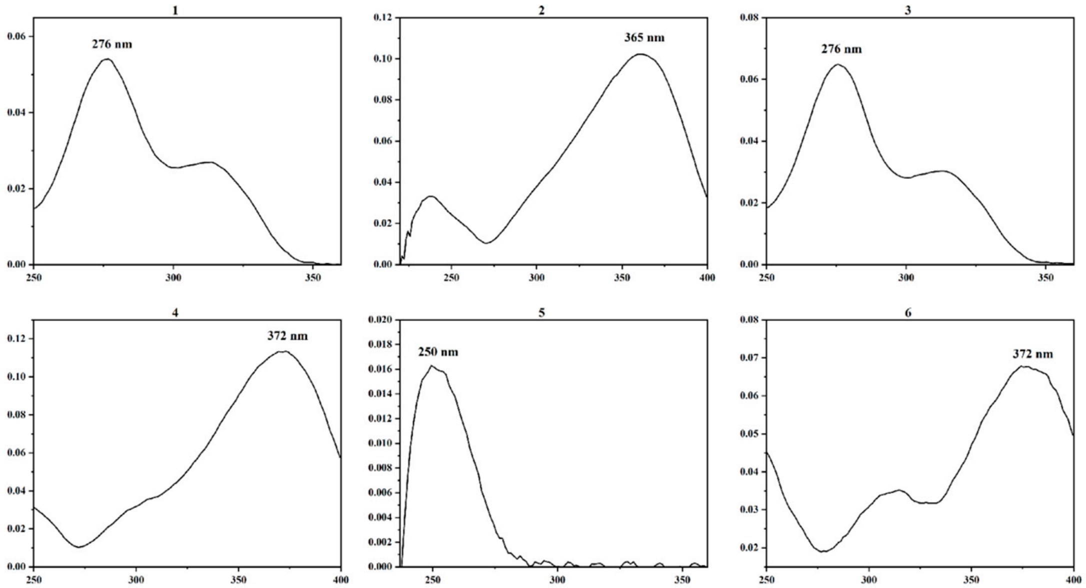

Supplemental Figure S2 UV-vis spectra of six components

(1: liquiritin; 2: isoliquiritin; 3: liquiritigenin; 4: isoliquiritigenin; 5: ammonium glycyrrhizinate; 6: licochalcone A)
